# Acute Hypoxic Stress Affects Migration Machinery of Tissue O_2_-Adapted Adipose Stromal Cells

**DOI:** 10.1155/2016/7260562

**Published:** 2016-12-28

**Authors:** Olga O. Udartseva, Margarita V. Lobanova, Elena R. Andreeva, Sergey V. Buravkov, Irina V. Ogneva, Ludmila B. Buravkova

**Affiliations:** ^1^Institute of Biomedical Problems, Russian Academy of Science, Khoroshevskoe Shosse 76A, Moscow 123007, Russia; ^2^Lomonosov Moscow State University, Faculty of Fundamental Medicine, Lomonosovsky Prospect 31-5, Moscow 117192, Russia; ^3^I.M. Sechenov First Moscow State Medical University, Trubetskaya Str. 8-2, Moscow 119991, Russia

## Abstract

The ability of mesenchymal stromal (stem) cells (MSCs) to be mobilised from their local depot towards sites of injury and to participate in tissue repair makes these cells promising candidates for cell therapy. Physiological O_2_ tension in an MSC niche in vivo is about 4–7%. However, most in vitro studies of MSC functional activity are performed at 20% O_2_. Therefore, this study focused on the effects of short-term hypoxic stress (0.1% O_2_, 24 h) on adipose tissue-derived MSC motility at tissue-related O_2_ level. No significant changes in integrin expression were detected after short-term hypoxic stress. However, O_2_ deprivation provoked vimentin disassembly and actin polymerisation and increased cell stiffness. In addition, hypoxic stress induced the downregulation of* ACTR3, DSTN, MACF1, MID1, MYPT1, NCK1, ROCK1, TIAM1*, and* WASF1* expression, the products of which are known to be involved in leading edge formation and cell translocation. These changes were accompanied by the attenuation of targeted and nontargeted migration of MSCs after short-term hypoxic exposure, as demonstrated in scratch and transwell migration assays. These results indicate that acute hypoxic stress can modulate MSC function in their native milieu, preventing their mobilisation from sites of injury.

## 1. Introduction

Multipotent mesenchymal stromal (stem) cells (MSCs) are defined as adult adherent nonhaematopoietic precursors that are able to differentiate into multiple lineages (adipogenic, osteogenic, and chondrogenic), express CD73, CD90, and CD105, and lack the expression of CD11b, CD14, CD34, and CD45 [1]. Because of their many unique features, these cells are attractive candidates for cell therapy. Studies in the last decade show that at least a part of MSCs are of perivascular origin [2–4] and can be isolated from many tissues (e.g., bone marrow, adipose tissue, and umbilical cord blood) [5]. Adipose tissue is currently considered the most abundant and accessible source of adult MSCs.

The majority of in vitro MSC studies are performed in a 95% air atmosphere (20% O_2_) whereas the physiological O_2_ level in their niche in vivo is about 4–7% [6, 7] and the areas of injury are characterised by severe hypoxia (less than 1% O_2_) [8]. In addition, numerous data indicate that O_2_ tension is one of the most important regulators of MSC functional activity [9, 10]. Particularly, we and other researchers have shown that the permanent expansion of MSCs under “physioxia” (tissue-related O_2_ values) stimulates proliferative activity, maintains MSCs in their undifferentiated state, and decreases their susceptibility to apoptotic stimuli [11–14]. These changes are accompanied by a shift of energy metabolism towards glycolysis [15] and upregulation of key “stemness” genes (i.e., Oct3/4, Sox2, and Nanog) [13]. Thus, the response of MSCs permanently cultured under ambient (20%) and physiological (5%) O_2_ to different factors (hypoxic stress particularly) may vary significantly.

One of the most important MSC characteristics is the ability to be mobilised from their tissue depot and to migrate to areas of tissue injury where they are involved in the regulation of an inflammatory response [16, 17] and tissue repair [18, 19]. In particular, it has been shown that MSCs are home to sites of inflammation and injury that are characterised by acute hypoxia and high concentrations of proinflammatory cytokines [20]. Although migration to areas of damage is essential for MSC-mediated repair and remodelling, we have only just begun to understand the mechanisms involved, and the effects of low O_2_ on targeted MSC migration remain poorly investigated. Raheja et al. showed that hypoxia is an important regulator of MSC recruitment [21]. Moreover, stabilisation of HIF-1*α*, the key transcriptional factor that mediates hypoxia-induced signal transduction, was found to modify RhoA activity [22, 23]. Therefore, hypoxia may represent one of the most important regulators of MSC motility and homing, which are essential for tissue regeneration and cell therapy. However, only a few studies have focused on the effects of O_2_ tension on MSC migration, and data on the effects of hypoxia on MSC motility are quite controversial [22, 24, 25].

The present study aimed to reveal the mechanisms underlying the reduction of ASC mobilisation from sites of injury and regeneration of tissue. To achieve this, we examined the effects of short-term hypoxic stress (0.1% O_2_) on the migration of adipose tissue-derived mesenchymal stromal cells (ASCs) permanently cultured under physiological O_2_ (5% O_2_).

## 2. Materials and Methods

### 2.1. Cell Isolation and Culture

Adipose tissue samples were obtained from the subcutaneous abdominal depots of patients undergoing dermolipectomy at the Souz Multidisciplinary Clinic (Moscow, Russia), as part of a scientific agreement. All procedures were approved by the Biomedicine Ethics Committee of the Institute of Biomedical Problems, Russian Academy of Sciences (Physiology Section of the Russian Bioethics Committee, Russian Federation National Commission for UNESCO, Permit #314/МCK/09/03/13). The samples were repeatedly rinsed in sterile phosphate-buffered saline (PBS) to remove contaminating debris and red blood cells. Then, adipose tissue was mechanically minced, and the isolation of stromal vascular fraction cells was performed as previously described [26]. Briefly, minced tissue pieces were enzymatically digested with 0.075% (equivalently 40.7 U/mL) collagenase type I (Sigma-Aldrich, USA) for 30 min at 37°C with agitation. Subsequently, the enzyme was inactivated with an equal volume of *α*-MEM (Gibco, Life Technologies, USA) containing 10% foetal bovine serum (FBS). Then the tissue was disintegrated by pipetting up and down and filtering through a 100 *μ*m nylon mesh. The stromal vascular fraction, containing the ASCs, was obtained by centrifuging the sample at 500 ×g for 10 min. The pellet was resuspended, and the cells were inoculated in *α*-MEM supplemented with 10% FBS, 50 U/mL penicillin, and 50 *μ*g/mL streptomycin (complete medium) under 5% O_2_ in a multigas CO_2_-incubator (Sanyo, Japan).

Twenty-four hours after plating, cells were carefully washed with PBS to remove nonadherent cells and debris. According to a joint statement of IFATS and ISCT, adherent cells are ASCs [27], and these cells were expanded until they reached 90% confluency. Then, cells were harvested and analysed for the expression of ASC surface antigens and multilineage differentiation potential (see below). Then, cells were replated at a density of 3000 cells/cm^2^ and maintained under 5% O_2_ in *α*-MEM supplemented as indicated above. ASCs from the 2nd to 4th passages and at 80–90% confluence were used for experiments. Hypoxic stress was induced for 24 h in a hypoxic chamber (Stem Cell Technology, USA), with the O_2_ concentration controlled by an O_2_ sensor and maintained at 0.1%.

Human peripheral blood mononuclear cells were isolated from blood samples collected from healthy individuals who had given their written informed consent. Cells were separated using density gradient centrifugation with Histopaque-1077 (Sigma, USA) [28] and maintained in RPMI-1640 (Gibco, Life Technologies, USA) supplemented with 5% heat-inactivated FBS, 50 U/mL penicillin, and 50 *μ*g/mL streptomycin. Mononuclear cells were stimulated with 10 *μ*g/mL phytohemagglutinin PHA-P (Sigma-Aldrich).

Cryopreserved human umbilical vein endothelial cell (HUVEC) samples were provided by the Cryocenter Cord Blood Bank (Moscow, Russia), as part of a scientific agreement. The cells were cultured in 199 medium (Gibco, Life Technologies, USA) supplemented with 10% FBS, 200 *μ*g/mL endothelial cell growth factor (Sigma-Aldrich), 2 mM glutamine (Gibco, Life Technologies, USA), 1 mM sodium pyruvate (Gibco, Life Technologies, USA), 50 U/mL penicillin, and 50 *μ*g/mL streptomycin under 20% O_2_ in a CO_2_-incubator (Sanyo, Japan). Proinflammatory activation of endothelial cells was induced by TNF*α* (10 ng/mL) (Sigma-Aldrich, USA) 24 h prior to cocultivation with ASCs.

### 2.2. Immunophenotyping of ASCs

Cell surface antigens were analysed by flow cytometry using a BD Accuri C6 cytometer (BD Biosciences, USA). Cells at 80–90% confluence were harvested by trypsinisation, washed with PBS, and incubated with mouse monoclonal primary antibodies. The minimal cell number was 10^5^ cells per test. The set of antibodies used in this study to characterise ASCs was based on the minimal surface marker panel (CD45, CD73, CD90, and CD105) proposed by the IFATS and ISCT [1, 27]. CD54/CD106 (BD Biosciences, USA) and CD25/CD69 (Beckman Coulter, France) were used as activation markers for HUVECs and lymphocytes, respectively.

### 2.3. In Vitro Differentiation Assay

ASCs of the 2nd to 4th passages were grown until 80–90% confluence. Then, cells were cultured in a complete growth medium supplemented with specific adipogenic (1 *μ*M dexamethasone, 0.5 mM IBMX, 10 *μ*g/mL insulin, and 100 *μ*M indomethacin) or osteogenic (0.2 mM ascorbic acid 2-phosphate, 10 mM glycerol 2-phosphate, and 0.1 *μ*M dexamethasone) inductors (Mesenchymal Stem Cell Adipogenesis and Osteogenesis Kits, Millipore, USA). The cells were fixed with 4% formaldehyde in PBS after 7 days of adipogenic or 21 days of osteogenic differentiation. Adipogenic differentiation was determined by staining intracellular lipid droplets with 0.5% Oil Red O solution. Osteogenic differentiation was identified by alizarin red S staining for mineralized matrix (Sigma-Aldrich, USA).

### 2.4. Cell Proliferation Assay

To evaluate cell proliferation, tissue O_2_-adapted ASCs before and after hypoxic stress were plated at an initial density of 3000 cells/cm^2^. Images of five randomly selected view fields (0.77 mm^2^ field area) were captured with a Nikon Eclipse Ti-U microscope (Nikon, Germany) at 0, 24, and 96 h after plating. The cells were counted using Image Analysis Software SigmaScan Pro 5.0 (SPSS Inc., USA). Population doubling (PD) times were calculated as

(1) PD=duration∗log⁡2log⁡final cell number−log⁡initial cell number.

See [29].

### 2.5. Cell Viability Assay

Cell viability was analysed using an Annexin V-FITC/PI Kit (Immunotech, France). Briefly, ASCs cultured at 5% O_2_ and with hypoxic stress (24 h) were harvested by trypsinisation and incubated with Annexin V-fluorescein isothiocyanate (FITC) and propidium iodide (PI) for 15 min in the dark at 4°C according to the manufacturer's instructions. Then, the cells were analysed using a BD Accuri C6 cytometer. Viable cells were defined as those negative for AnnexinV-FITC and PI staining, apoptotic cells were defined as those positive for Annexin V-FITC and negative for PI staining, and necrotic cells were defined as those positive for PI staining.

### 2.6. Detection of Reactive Oxygen Species (ROS) and NO

Total of ROS and NO was detected using CM-H2DCFDA (a chloromethyl derivative of H2DCFDA) (Molecular Probes Inc., USA) and DAF-FM diacetate (Molecular Probes Inc., USA) according to manufacturer's instructions. Briefly, ASCs at 80–90% confluence were incubated in culture plates with 10 *μ*M CM-H2DCFDA or 2 *μ*M DAF-FM diacetate for 20 min at 37°C under 5% or 0.1% O_2_. After washing with PBS, cells were harvested and analysed by flow cytometry (*Ex*/*Em* = 490/530 nm). Intracellular ROS and NO levels were evaluated by measurements of chloromethyl-dichlorofluorescein (chloromethyl-DCF) or benzotriazole fluorescence after oxidation of CM-H2DCFDA or nitrosylation of DAF-FM diacetate, respectively.

### 2.7. In Vitro Wound-Healing Assay

Nontargeted ASC migration was evaluated in cell monolayers at a cell density of 10^4^ cells per cm^2^ using the in vitro “scratch” assay [30]. A confluent monolayer was scratched with a sterile pipette tip to create a “wound” approximately 0.8–1.0 mm wide. Then, culture medium was replaced with *α*-MEM supplemented with 10% FBS to remove cell debris. All scratch assays were performed in six replicates. To estimate the wound closure, serial digital images were captured with a Nikon Eclipse Ti-U microscope (Nikon, Germany) immediately after and at specific time intervals (3, 6, 9, and 24 h) after the scratch. The images were analysed using NIS-Elements software (Nikon, Germany) to measure the width of the scratch at previously marked points (five per Petri dish) along its length. The migration area was calculated as the difference between the initial and final wound squares.

### 2.8. Transwell Migration Assay

The transwell assay assesses not only migratory activity but also chemotactic effects. Therefore, in present study, migration assays were performed in 24-well transwell plates (Corning Costar, USA) using polycarbonate membranes with 8 *μ*m pores (Corning Costar, USA). Proinflammatory activated endothelial cells and PBMCs were the targets for ASC migration. ASCs at a density of 5 × 10^4^ cells/mL in 150 *μ*L of medium were placed in the upper chamber of the transwell assembly. The lower chamber contained 600 *μ*L of phytohemagglutinin-activated mononuclear suspension (5 × 10^5^ cells/mL) or monolayers of TNF*α*-activated endothelial cells. After 24 h of incubation, the upper surface of the membrane was scraped gently to remove nonmigrating ASCs and washed with PBS. The membrane was then fixed in 4% paraformaldehyde for 15 minutes and stained in 1 M ethidium bromide (Sigma-Aldrich, USA) for 10 minutes. The number of migrating cells was determined by counting ten random view fields per well using a Nikon Eclipse Ti-U microscope.

### 2.9. Fluorescent Staining

ASCs were grown on coverslips. For immunofluorescent and phalloidin staining, cells were fixed with 4% formaldehyde in PBS for 15 min and permeabilised with 0.1% Triton X-100 for 10 min. Then, the cells were washed with PBS 3 times and incubated with the primary anti-vimentin mouse monoclonal antibody (Chemicon, Millipore, USA) or anti-tubulin mouse monoclonal antibody (Santa Cruz Biotechnology, USA) for 1 h at 37°C. Subsequently, rhodamine phalloidin (Molecular Probes, Life Technologies, USA) and Alexa Fluor 488-conjugated anti-mouse IgG secondary antibodies (Molecular Probes, Life Technologies, USA) were added for 1 h. Then, the samples were washed and mounted with Fluoroshield with DAPI (Sigma-Aldrich). Images were acquired using an LSM 780 (Carl Zeiss, Oberkochen, Germany) confocal microscope.

### 2.10. Atomic Force Microscopy

Atomic force microscopy is a useful tool for studying cell mechanics [31]. In this study, cell transversal stiffness was measured using a Solver P47-Pro instrument (NT-MDT, Moscow, Russia) as previously described [32]. Briefly, ASCs were grown on round glass coverslips (12 mm diameter) for cell culture (Corning, USA). Then, coverslips with ASC monolayers were mounted onto the liquid cell of the atomic force microscope adjusting table. Force-distance curves were obtained in contact mode using a soft silicon cantilever. The cantilever spring constant (Microlever, Park Scientific Instruments, USA) and radius of the tip curvature were 0.01 N/m and 10 nm, respectively. The actual indentation depth and force applied were calculated using the following formula: *h*_*s*_ = *x* − *y* · *a*, *F*_*s*_ = *y* · *a* · *k*_*c*_, where *h*_*s*_ is the actual indentation depth (m), *F*_*s*_ is the actual force applied to a cell (N), and *k*_*c*_ is the cantilever stiffness coefficient. At an indentation depth of 150 nm, the change in applied force was determined and cell stiffness was estimated using the following formula: *k*_*s*_ = *F*_*s*_/*h*_*s*_. The results were processed using MATLAB 6.5 software developed especially for this research.

### 2.11. Protein Extraction and Western Blotting

Cells were lysed with a lysis buffer containing 63 mM Tris-HCl, 10% glycerol, 5%  *β*-mercaptoethanol, 2% SDS (pH 6.8), and Halt Protease Inhibitor Cocktail (ThermoFisher Scientific, USA) on ice. The protein concentration of lysates was measured using a Nanodrop 2000c (ThermoFisher Scientific, USA), and equal amounts of the protein were loaded into the wells of an SDS-polyacrylamide gel electrophoresis (PAGE) gel (10%). Then, proteins were separated by SDS-PAGE and transferred onto nitrocellulose membranes according to standard protocols at 90 mA overnight (+4°C). For the detection of specific proteins, the following primary and secondary antibodies were used: anti-*β*-actin (1 : 200), anti-*ϒ*-actin (1 : 200), anti-*β*-tubulin (1 : 200), anti-vinculin (1 : 50) (Santa Cruz Biotechnology, USA), and anti-mouse IgG-biotin antibodies (1 : 2000) (Sigma-Aldrich, USA). After incubation with secondary antibodies, all membranes were treated with streptavidin-peroxidase (1 : 4000) (Sigma-Aldrich, USA). Specific protein bands were detected using 3,3′-diaminobenzidine (Sigma-Aldrich, USA) and analysed with ImageJ software. Protein levels were estimated by densitometry and normalised with respect to tubulin used as a loading control.

### 2.12. Quantitative RT-PCR Analysis

Expression of 252 genes after hypoxic stress was analysed using a Cytoskeleton Regulators RT^2^ Profiler PCR Array, PI3K-AKT Signalling Pathway RT^2^ Profiler PCR Array, and MAP Kinase Signalling Pathway RT^2^ Profiler PCR Array (Qiagen, USA).

To evaluate gene expression, total RNA was extracted with QIAzol Reagent (Qiagen, USA) and purified by the phenol/chloroform technique. Reverse transcription was performed using a QuantiTect Reverse Transcription Kit (Qiagen, USA) according to the manufacturer's protocol. Resulting cDNA was mixed with RT^2^ SYBR Green/ROX PCR Master Mix (Qiagen, USA) and added to 96-well plates. PCR was performed using the Mx300P system (Stratagene, USA), and the obtained data were analysed using RT^2^ Profiler PCR Array Data Analysis ver. 3.5 software (http://pcrdataanalysis.sabiosciences.com/pcr/arrayanalysis.php). The expression levels of five housekeeping genes (*ACTB*,* B2M*,* GAPDH*,* HPRT*, and* RPLP0*) included in the arrays were profiled. Based on their stable expression under hypoxic conditions,* B2M, HPRT*, and* RPLP0 *were selected as reference genes for normalisation of target gene expression. Normalised gene expression was calculated by the 2^−ΔΔCt^ method. *p* values were calculated based on Student's *t*-test for the replicate 2^−ΔΔCt^ values for each gene in the control and treatment groups.

### 2.13. Statistical Analysis

All data were calculated from 3–5 independent experiments and expressed as the mean ± standard deviation. Analysis of group differences was performed by Mann–Whitney *U* test for independent samples using SPSS 14.0 software. Statistical significance was considered at *p* < 0.05.

## 3. Results

### 3.1. Characterisation of Tissue O_2_-Adapted ASCs under Different O_2_ Conditions

As the O_2_ level routinely used in cell culture (20% O_2_) did not reflect physiological conditions, we analysed the effects of acute hypoxic stress (0.1% O_2_ for 24 h) on the characteristics of ASCs maintained under 5% O_2_.

**Figure 1:**
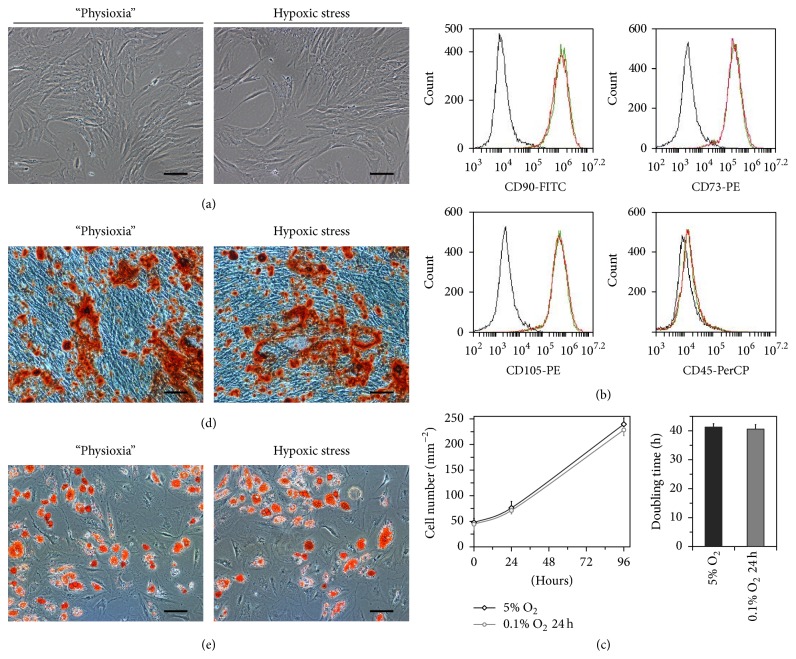
Adipose tissue-derived mesenchymal stromal cells (ASCs) under physioxia (5% O_2_) and short-term hypoxic stress (0.1% O_2_, 24 h). (a) Phase-contrast microscopy of ASCs showed no morphological differences (scale bar, 100 *μ*m). (b) The phenotype of ASCs was identified by flow cytometric analysis. ASCs were positive for CD73, CD90, and CD105 and negative for CD45. The black line indicates the isotype control, the green line indicates tissue O_2_-adapted ASCs, and the red line indicates ASCs after short-term hypoxic stress. (c) Cell growth was evaluated by growth curves and population doubling time over 4 days. The data are presented as the mean ± standard deviation (*n* = 3). (d) Osteogenic and (e) adipogenic induction was assessed by alizarin red S staining of the mineralized matrix and Oil Red O staining of intracellular neutral lipid droplets, respectively (scale bar, 100 *μ*m).

The morphology of cells permanently cultured at physioxia and exposed to acute hypoxia was revealed to be similar (Figure 1(a)). Flow cytometric analysis of ASCs demonstrated that hypoxic stress did not alter the expression of stromal CD markers, and the ASCs of both groups were found to be positive for CD73 (97.67 ± 2.52%), CD90 (99.57 ± 0.51%), and CD105 (98.63 ± 1.52%) and negative for CD45 (0.2 ± 0.1%) (Figure 1(b)). Evaluation of cell growth in both ASC groups revealed no intergroup differences in cell number increase and population doubling time (Figure 1(c)), suggesting that short-term hypoxic stress did not affect ASC proliferative activity in vitro. Moreover, acute hypoxic stress affected neither the osteogenic nor adipogenic potential of ASCs (Figures 1(d) and 1(e)).

### 3.2. Intracellular ROS Level and ASC Viability after Short-Term Hypoxic Stress

**Figure 2:**
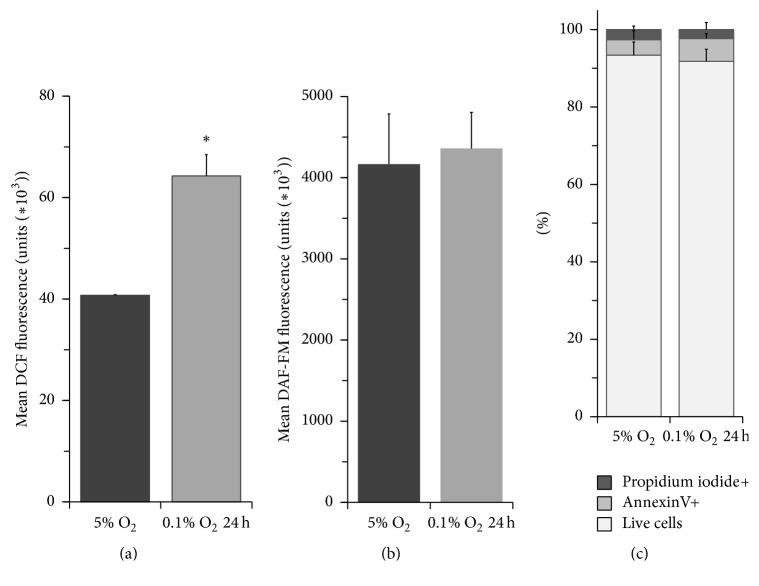
Hypoxic stress induces intracellular ROS but does not affect adipose tissue-derived mesenchymal stromal cell viability. (a) Intracellular ROS were detected with CM-H_2_DCFDA (10 *μ*M for 20 min) and analysed by flow cytometry. (b) Intracellular NO levels were evaluated by flow cytometry using DAF-FM diacetate (2 *μ*M for 30 min, followed by a 30 min incubation in fresh medium for complete deesterification). (c) Cell viability was determined by analysing AnnexinV and propidium iodide binding by flow cytometry. Data represent the mean + standard deviation (*n* = 3), ^*∗*^ *p* < 0.05.

We examined intracellular ROS and NO levels in ASCs after short-term hypoxic stress using CM-H2DCFDA and DAF-FM diacetate, respectively, because ROS may be a regulator of hypoxia-mediated signal transduction [33, 34]. Short hypoxic exposure was shown to result in a 1.5-2-fold increase in intracellular ROS, whereas NO production remained unchanged under these conditions (Figures 2(a) and 2(b)). At the same time, the viability of ASCs cultured at 5% O_2_ was high (93.4 ± 3.4%), and no cell death induction was detected after acute hypoxic stress (91.8 ± 3.1%) (Figure 2(c)).

### 3.3. Short-Term Hypoxic Stress Attenuates “Wound-Healing” Potential and Targeted Migration of ASCs

**Figure 3:**
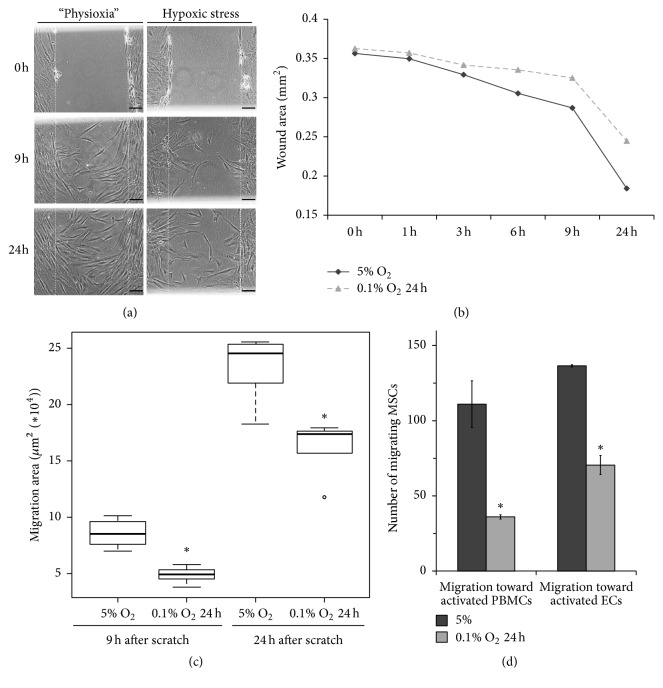
Adipose tissue-derived mesenchymal stromal cell (ASC) migration in scratch ((a)–(c)) and transwell (d) assays. (a) Representative phase-contrast images of the in vitro wound-healing assay (scale bar, 100 *μ*m). Monolayers of cells cultured at 5% O_2_ or after short-term hypoxic stress (0.1% O_2_, 24 h) were scraped, and images were taken at 0, 1, 3, 6, 9, and 24 h. The dotted lines define the areas of scratches. (b) Representative curves of time-dependent wound closure (*n* = 5). (c) Statistical analysis of migration results (^*∗*^ *p* < 0.05, *n* = 5). (d) Targeted migration of ASCs was analysed in transwell assay. The tissue O_2_-adapted ASCs before and after hypoxic stress were seeded on a membrane in the upper chamber and allowed to migrate for 24 h towards phytohemagglutinin-activated peripheral blood mononuclear cells (PBMCs) or TNF*α*-activated endothelial cells (ECs) cultured in the lower chamber. The migration rate was defined as a number of migrated ASCs (^*∗*^ *p* < 0.05, *n* = 3).

Nontargeted ASC migration was analysed by scratch (wound-healing) assay. To evaluate the effects of acute hypoxic stress, cells cultured under physioxia (5% O_2_) were exposed to short-term O_2_ deprivation (0.1% O_2_ for 24 h), followed by scratching of the monolayer (Figure 3(a)). Wound closure was estimated 3, 6, 9, and 24 h after the scrape (Figure 3(b)). Short-term hypoxic stress was demonstrated to result in a 1.5-fold decrease in ASC motility and wound-healing potential (Figure 3(c)).

Targeted ASC migration under hypoxic conditions was analysed using a modified Boyden chamber migration assay with Transwell® culture plates. We found that hypoxic stress considerably affected the migration of tissue O_2_-adapted ASCs towards activated endothelial cells and lymphocytes. The number of migrating cells after acute hypoxic exposure was more than two times less than the number of ASCs migrating at 5% O_2_ (Figure 3(d)). These results showed that hypoxic stress decreases not only cell motility, but also the ability of ASCs to respond to chemotactic stimuli.

### 3.4. Hypoxic Stress Leads to Alterations in F-Actin and Vimentin, but Not Tubulin Structures

**Figure 4:**
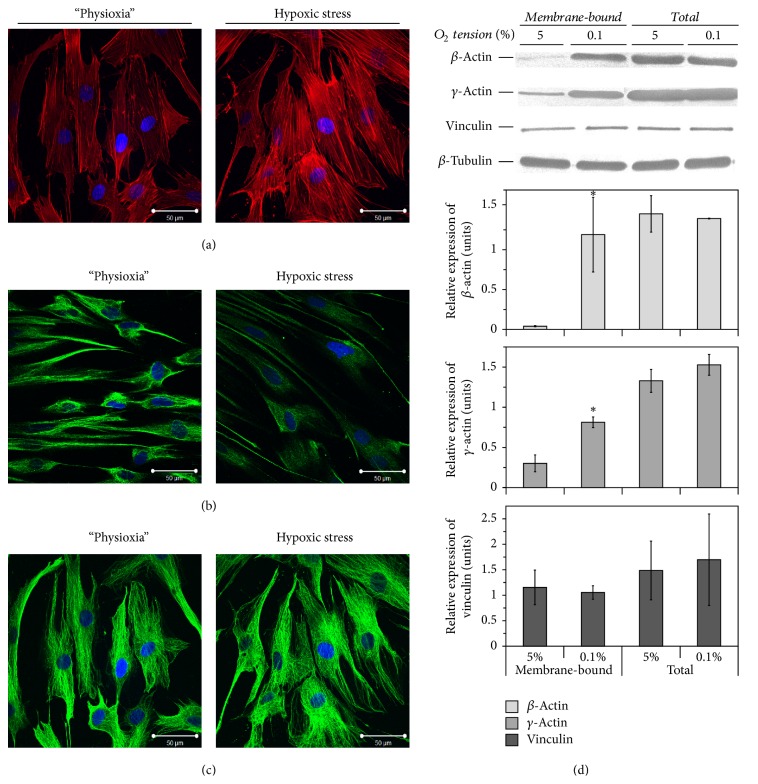
The effects of hypoxic stress on cytoskeleton organisation of tissue O_2_-adapted adipose tissue-derived mesenchymal stromal cells (ASCs). ((a)-(c)) Representative images of phalloidin staining (a) and immunofluorescent labelling of ASCs for vimentin (b) and *β*-tubulin (c) before and after hypoxic stress (0.1% O_2_, 24 h) (*n* = 3). Scale bar is 50 *μ*m. (d) Expression of *β*-, *γ*-actin, *β*-tubulin, and vinculin in total and membrane protein extracts obtained from ASCs before and after short-term hypoxic stress (mean + standard deviation, ^*∗*^ *p* < 0.05, *n* = 3).

Cytoskeleton organisation is one of the most important factors that determine cell motility. Therefore, we studied the effects of hypoxia on cell filament organisation. Acute hypoxic stress was shown to result in an obvious change in the actin cytoskeleton and intermediate filament structure but to have no significant effect on the structure of microtubules (Figure 4(a)). Under physioxia, intermediate filaments of ASCs formed nest-like structures, whereas hypoxia preconditioning promoted vimentin depolymerisation (Figure 4(a)). Sparse, randomly arranged actin fibrils were typical for ASCs cultured under 5% O_2_, whereas hypoxic stress stimulated actin polymerisation, which was manifested by a thickening of and increase in the number of parallel microfibrils and formation of F-actin assemblies (aggregates) (Figure 4(a)). Furthermore, we found that short-term exposure of ASCs to 0.1% O_2_ increased the amount of membrane-bound, but not total, *β*- and *γ*-actin and did not affect the amount of vinculin and *β*-tubulin (Figure 4(b)).

### 3.5. Acute Hypoxic Exposure Enhances ASC Stiffness

**Figure 5:**
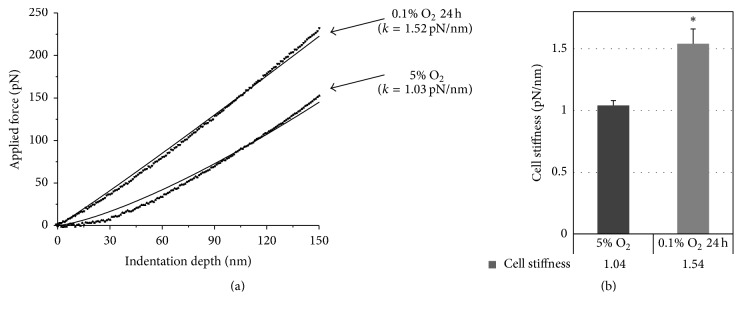
Adipose tissue-derived mesenchymal stromal cells (ASCs) stiffness under physioxia (5% O_2_) and hypoxic stress (0.1% O_2_, 24 h). (a) Representative force curves, obtained during measurements of cell stiffness. (b) Hypoxic stress increased cell stiffness (mean + standard deviation, ^*∗*^ *p* < 0.05, *n* = 3). Seventy cells were analysed in each group.

Based on the observation that acute hypoxia affects ASC migratory potential and cytoskeleton organisation, we used atomic force microscopy to evaluate the effect of hypoxic stress on cell stiffness. We found that short-term exposure of ASCs to 0.1% O_2_ resulted in a 1.5-fold increase in their stiffness (Figure 5).

### 3.6. Effects of Acute Hypoxia on Expression of Adhesion Molecules

**Figure 6:**
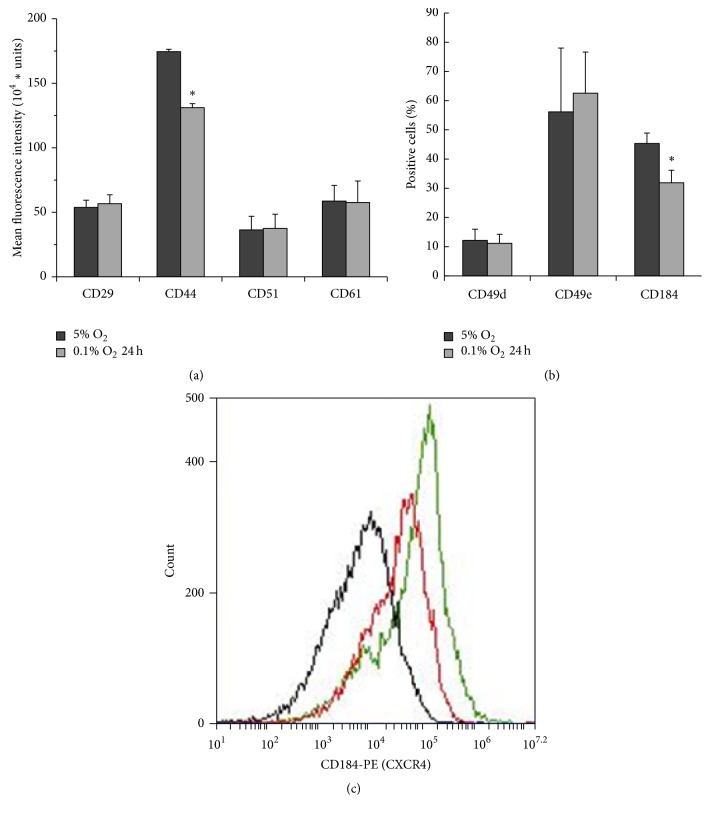
Comparative analysis of the expression of adipose tissue-derived mesenchymal stromal cell (ASC) surface antigens under physioxia and hypoxic stress. Flow cytometric data are presented as (a) mean fluorescence intensity of antigen staining (100% of ASCs were positive) or (b) the percentage of positively stained cells (mean + standard deviation, ^*∗*^ *p* < 0.05). (c) The surface expression of CXCR4 on ASCs under hypoxic stress (0.1% O_2_). The black line indicates the isotype control, the green line indicates tissue O_2_-adapted ASCs, and the red line indicates ASCs after short-term hypoxic stress. Data are representative of three independent experiments.

Expression of adhesion molecules is another factor that regulates the functional properties of ASCs and their migration capacities. In this study, we used flow cytometry to analyse the effects of short-term hypoxic stress on the expression of CD29 (integrin *β*1), CD44 (HCAM), CD49d (integrin *α*4), CD49e (integrin *α*5), CD51 (integrin *α*V), CD61 (integrin *β*3), and CD184 (CXCR4). All ASCs cultured at 5% O_2_ were positive for CD29, CD44, CD51, and CD61 (Figure 6), whereas only a part of cells were positive for CD49d, CD49e, and CD184. We found out that short-term exposure of ASCs to 0.1% O_2_ affected HCAM and CXCR4 expression. In particular, hypoxic stress reduced not only the percentage of cells expressing the marker (Figure 6(b)), but the expression of CXCR4 itself too, as indicated by decrease of mean fluorescent intensity (Figure 6(c)). In addition, acute hypoxic stress impaired CD44 expression, whereas no significant changes in integrin expression were found between physioxia-cultured and hypoxia-exposed ASCs.

### 3.7. Expression of Akt-, MAPK-, and Cytoskeleton-Related Genes under Acute Hypoxic Stress

**Figure 7:**
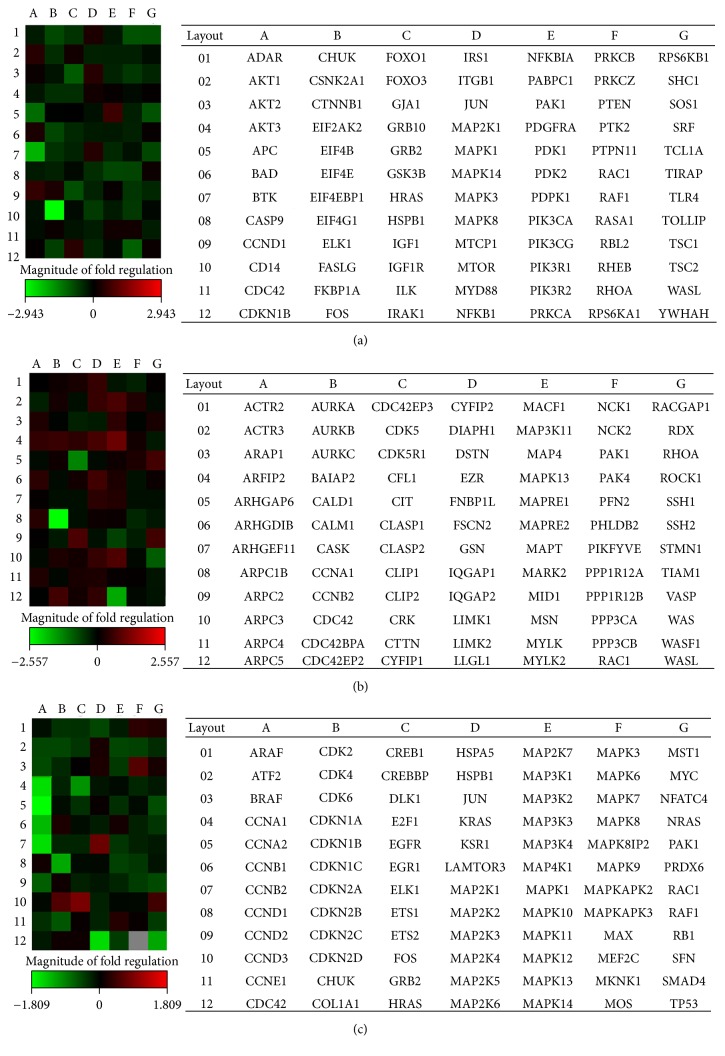
Differential gene expression. Heat maps display the gene expression changes in 84 PI3K-Akt-related (a), cytoskeleton-related (b), or MAPK-related (c) genes in tissue O_2_-adapted adipose tissue-derived mesenchymal stromal cells (ASCs) after short-term hypoxic stress. Gradients indicate the level of gene expression change.

No dramatic changes in gene expression were detected after short-term ASC exposure to 0.1% O_2_. However, expression of 36 of the 252 analysed genes was shown to vary by more than 1.5 times with acute hypoxic stress (Figure 7 and Table 1). At the same time, the expression of seven genes (*APC, CCNA1, FASLG, FOS, GJA1, MAP2K6*, and* MID1*) showed more than a 2-fold change. Seven genes only (*FOS, CDKN2D, MAP2K1, MAPK7, MAPK13, PDK1*, and* SFN*) were characterised by an increase in their expression, whereas the expression of other genes was reduced. Analysis of gene expression of cytoskeleton regulators indicated that short-term exposure of ASCs to 0.1% O_2_ downregulated the expression of* ACTR3, DSTN, MACF1, MID1, MYPT1, NCK1, ROCK1, TIAM1, *and* WASF1,* the products of which represent important regulators of *β*-actin depolymerisation and lamellipodia formation. The downregulation of* p53 *and upregulation of stress-induced* SFN* and* p38-delta* expression detected here may contribute to ASC survival under acute hypoxic stress.

**Figure 8:**
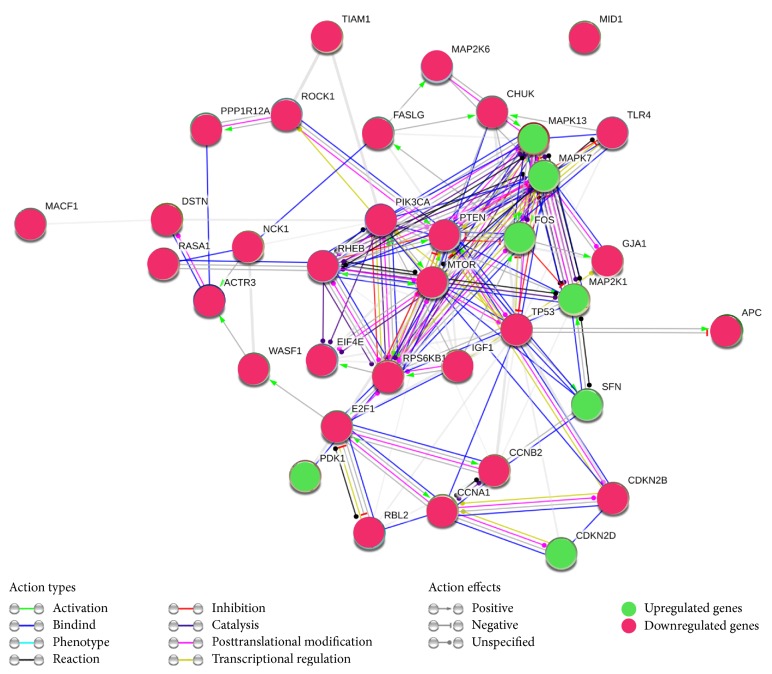
Interactive network of migration-related genes altered under short-term hypoxic stress. The database STRING 9.1 was used to find and visualise predicted protein-protein interactions.

An interactive network of migration-related genes that showed altered expression under hypoxic stress is presented in Figure 8. This interactive network was built using the STRING 9.1 database and demonstrates the predicted interaction of proteins encoded by migration-related genes with variable expression. We suppose that components of the MAPK- and PI3K/mTOR/PTEN-signalling cascades (FOS, MAPK7, MAPK13, MAP2K1, MTOR, PI3CA, PTEN, RHEB, RPS6KB1, and TP53) play an important role in cell motility [35, 36] and represent functional modules of this interactive network. The other components of the network (ACTR3, DSTN, MACF1, MID1, MYPT1, NKC1, TIAM1, and WASF1) are structural proteins or effectors, but are not key regulators of cell function, and their protein interactions are poorly understood. Therefore, there are only a few known links between these proteins and the principal network of MAPK- and Akt-signalling molecules.

## 4. Discussion

In the present study, acute hypoxic stress was shown to significantly impair the migration ability of tissue O_2_-adapted ASCs. This was accompanied by actin and vimentin reorganisation and downregulation of some PI3K-, MAPK-, and cytoskeleton-related genes.

There is a growing number of published reports comparing features of cells cultured at different O_2_ concentrations. There is no doubt that the level of O_2_ in culture medium is an important factor controlling the functional activity of cells. O_2_ concentrations are known to vary from 1 to 10% (physioxia) in different tissues [7]. In this regard, we and many other researchers consider standard cell culture conditions (20% O_2_) to be mild hyperoxia, characterised by increased activity of the antioxidant defence system enzymes [37, 38].

Various pathologic conditions (ischemia, inflammation, tumours, and tissue injury) are followed by local reductions in the tissue O_2_ partial pressure to 0.1% [39]. MSCs are among the key players in tissue reparation. Thereby, the effects of permanent hypoxia and acute hypoxic stress on the functional characteristics of MSCs have been the focus of extensive research [15, 25, 40, 41]. However, publications regarding the cellular response to a hypoxic state are contradictory. Most of the discrepancies can be explained by differences in O_2_ concentration, exposure time, and cell sources. In general, there is increasing evidence of mild hypoxia acting as a potent regulator of various stem cell types [9, 42]. O_2_ tension over 1% acts as a proliferative stimulus for most cell types and prevents cell cycle arrest, whereas anoxia and very low O_2_ level (less than 1%) induce MSC cell stress and apoptosis [12, 13, 43, 44]. Thus, the effects of low oxygen level on stem cells are extensive and depend on severity of hypoxia.

Here, we found that short-term hypoxic exposure does not affect ASC minimal criteria parameters and viability, but that it significantly impairs targeted and nontargeted migration of tissue O_2_-adapted ASCs. The maintenance of high cell viability after hypoxic stress can be explained by at least two of our observations. Firstly, the increased level of intracellular ROS at 0.1% O_2_ may enhance the activity of ASC antioxidant systems. Previously, we demonstrated mild oxidative stress followed by an increase in superoxide dismutase activity in ASCs cultured continuously under 5% O_2_ [38, 45]. Secondly, the downregulation of* TP53* (p53),* FASLG* (Fas ligand), and* APC* gene expression and upregulation of* SFN* (14-3-3 sigma) expression found in this study should prevent apoptotic events. In studies of foetal and bone marrow MSCs, Fas ligand was shown to play an important role in the regulation of not only immunocompetent but also stem cell apoptosis [46, 47]. Although little data on the impact of hypoxia on MSC gene regulation are available, studies on other cells (HCT116) have shown that 14-3-3 sigma (encoded by* SFN* gene), a p53-induced G_2_/M check-point protein, enhanced cell survival under stress as a result of Bax sequestration [48]. In the HT-29 cell line, the induction of APC expression was found to result in a 10-fold increase in the number of apoptotic cells because of an increase in caspase activity [49, 50].

Cytoskeleton organisation is the major regulator of cell motility. Previously, cell motility was found to be related to cell stiffness [51], and the dominant feature controlling cellular stiffness appears to be the F-actin cytoskeleton [52]. Our data show that the stabilisation of F-actin and stress fibre formation in ASCs under hypoxic stress is accompanied by increased cell stiffness. This agrees with recent studies that have revealed a correlation between low cell stiffness and high motility/metastatic potential of cancer cells [53, 54]. This phenomenon can be explained in several ways. Firstly, highly motile cells (i.e., cancer cells) are known to have decreased stress fibres, increased cortical actin, and more lamellipodia, which are much softer than stress fibres [55]. Secondly, highly motile cells are characterised by low adhesion to substrate and a high ratio of focal adhesion turnover [56]. According to our data, we suppose that hypoxic stress promotes the stabilisation of focal adhesions and formation of focal adhesion plaques at the end of stress fibres and results in an increase in membrane-bound actin.

MSC migration regulatory mechanisms under hypoxia are still poorly understood, despite the fact that the ability of MSCs to migrate to areas of damage is a key parameter determining these cells' potential for use in cell therapy. Formation of the “leading edge,” based on actin polymerisation regulated by a complicated protein complex of Arp2/3, DSTN, WASF, profilin, cofilin, and other proteins, is known to be the first step in directional cell translocation [57, 58]. Further development of the active edge and forward cell translocation are only possible with the formation of adhesive structures (focal complexes) in the active edge area, which results in reorganisation of the actin filament network, F-actin assembly formation, and the onset of contractile tension required for cell translocation. Small GTPases of the Rho family (Rho, Rac, and CDC42) play a central role in the regulation of actin-binding proteins, cytoskeleton dynamics, and cell motility [59]. Numerous studies of various cell types have shown that HIF-1*α* stabilisation has a considerable effect on RhoA activity, which is followed by changes in migration activity [22, 23, 60]. We have previously demonstrated changes in the expression of HIF family transcription factors in tissue O_2_-adapted ASCs after hypoxic stress [45]. Particularly, statistically significant downregulation of* HIF-1α*and upregulation of* HIF-3α*expression in ASCs were found 24 h after hypoxic (1% O_2_) exposure. These data well correlate with studies of other groups who demonstrated that activation of HIF-1*α* is detectable at earlier time point (between 4 and 8 hours), and then HIF-1*α* disappears [61]. In present study, we failed to detect effects of hypoxic stress on the transcription of* Rho, Rac,* and* CDC42* genes. However, the observed increase in the number and thickness of stress fibres may indicate increased RhoA activity under acute hypoxic stress (0.1% O_2_), which is consistent with the findings of other studies [23, 62]. ROCK (Rho kinase) is the main Rho effector that targets intermediate filament proteins [59]. Therefore, we proposed that vimentin disassembly under hypoxic stress was the result of RhoA and ROCK activation. As mentioned above, stress fibril formation is insufficient to provide directional cell translocation, for which formation of lamellipodia and a “contractile” cell apparatus are required. However, we showed in this study that ASC exposure to low O_2_ results in the reduced expression of* ACTR3, DSTN, NCK1,* and* WASF1 *genes, the products of which are actin-binding proteins involved in active edge formation [58, 63, 64]. The downregulation of* MACF1, MID1,* and* MYPT1 *genes, the products of which are involved in the formation of actomyosin complexes that provide the contractive tension required for the translocation of migrating cells, was detected. Thus, we assume that the inhibition of ASC migration activity under hypoxic stress is associated with their increased stiffness, decreased ability to form leading edges, and decreased contractility because of the dissociation of actomyosin complexes.

The targeted migration of cells (chemotaxis) is regulated by chemokines and their receptors, whose interaction activates signalling cascades resulting in cytoskeleton remodelling. A number of studies have shown SDF-1 (CXCL12) and PDGF-B to be the main chemoattractants regulating MSC mobilisation, trafficking, and homing [20, 65, 66]; their chemokine receptors are CXCR4 (CD184) and PDGFR-*β*, respectively. Furthermore, it is known that integrin-extracellular matrix interaction may activate PDGFR-*β* in the absence of growth factors and stimulate cell migration [67, 68]. In this study, we showed that hypoxic stress reduces the ability of ASCs to migrate towards activated endothelial and peripheral blood mononuclear cells. In addition, we found no effects of reduced O_2_ concentration (0.1%) on the expression of integrins but showed that short-term ASC exposure to acute hypoxia reduced the expression of CXCR4 and HCAM-1 (CD44). A number of studies have demonstrated an important role for chemokine receptor CXCR4 in MSC mobilisation and chemotaxis, with its neutralisation reducing homing and engraftment of MSCs significantly [69–71]. We suppose that a decrease in CXCR4 (CD184) expression is one of the mechanisms involved in the regulation of tissue O_2_-adapted targeted ASC migration under hypoxic stress. Moreover, a decrease in CXCR4 (CD184) expression can result in a reduction of ASC mobilisation from sites of injury, which may play an important role in tissue regeneration. In addition, recent studies have confirmed an important role for hyaluronic acid receptor (CD44) in the regulation of MSC migration towards the area of damage [72]. In this regard, we can assume that the observed reduction in ASC capability of chemotaxis is CXCR4- and CD44-dependent.

To the best of our knowledge, these are the first results to describe the effects of acute hypoxic stress on the motility of MSCs permanently cultured under tissue-related O_2_. Hypoxic inhibition of progenitor cell migration may serve several functions. Firstly, acute hypoxia in the MSC microenvironment indicates local ischemia and the need for tissue vascularisation and repair. This suggestion is indirectly confirmed by our previous data on the paracrine activity of tissue-adapted O_2_ ASCs under acute hypoxia. In addition, we have demonstrated that hypoxic stress stimulates the secretion of proangiogenic factors (VEGF, IL-8) from tissue O_2_-adapted ASCs [45], which should promote angiogenesis. The above results correlate with the findings of other studies, confirming that hypoxic preconditioning promotes MSC angiogenic activity [41, 73]. Secondly, the release of chemoattractants from cells in the areas of damage creates a chemotactic gradient that stimulated MSC migration from perivascular niches towards the areas of injury, which are characterised by acute hypoxia. It is also possible that the inhibition of MSC migration under acute hypoxia contributes to MSC protection by delaying their migration until O_2_ concentrations are compatible with cell survival. Further research into therapeutic enhancement of angiogenesis and O_2_ delivery may serve to speed the repair process and aid in the reconstitution of damaged tissues.

## 5. Conclusions

In the present study, we have shown that acute hypoxic stress attenuates tissue O_2_-adapted ASC motility and wound-healing potential. Moreover, the response of ASCs to chemotactic stimuli depends significantly on the O_2_ level in the microenvironment. Changes in tissue O_2_-adapted ASC motility under hypoxic stress are accompanied by cytoskeleton reorganisation, increased cell stiffness, and decreased expression of HCAM and CXCR4, but not integrin. Our results highlight the importance of O_2_ in the regulation of stromal progenitor cell function in the areas of ischemic tissue damage. However, the above results diverge from the data obtained on MSCs under “normoxia” (20% O_2_), which should be taken into consideration when discussing MSC involvement in tissue regeneration. Further mechanisms that mediate hypoxia-induced signal transduction in physioxic MSCs should be investigated.

## Figures and Tables

**Table 1:** Differential gene expression in tissue O_2_-adapted ASCs after short-term hypoxic stress.

Gene name	Description	Fold regulation	*p* value
*PI3K-AKT signalling pathway*			

*APC*	*Adenomatous polyposis coli*	*−2,37*	*0,001822*
*CHUK*	Conserved helix-loop-helix ubiquitous kinase	−1,77	0,002
*EIF4E*	Eukaryotic translation initiation factor 4E	−1,75	0,025246
*FASLG*	*Fas ligand (TNF superfamily, member 6)*	*−7,69*	*0,027667*
*GJA1*	*Gap junction protein, alpha 1, 43 kDa*	*−2,05*	*0,000032*
*IGF1*	Insulin-like growth factor 1 (somatomedin C)	−1,86	0,000335
*MTOR*	Mechanistic target of rapamycin (serine/threonine kinase)	−1,58	0,00017
*PDK1*	Pyruvate dehydrogenase kinase, isozyme 1	1,62	0,00023
*PIK3CA*	Phosphoinositide-3-kinase, catalytic, alpha polypeptide	−1,76	0,000144
*PTEN*	Phosphatase and tensin homolog	−1,55	0,000564
*RASA1*	RAS p21 protein activator 1	−1,72	0,000345
*RBL2*	Retinoblastoma-like 2 (p130)	−1,53	0,000685
*RHEB*	Ras homolog enriched in brain	−1,56	0,001571
*RPS6KB1*	Ribosomal protein S6 kinase, 70 kDa	−1,91	0,000001
*TLR4*	Toll-like receptor 4	−1,75	0,000149

*Cytoskeleton regulators*			

*ACTR3*	ARP3 actin-related protein 3 homolog	−1,68	0,007654
*CCNA1*	Cyclin A1	*−3,56*	*0,001399*
*CCNB2*	Cyclin B2	−1,58	0,03013
*DSTN*	Destrin (actin depolymerising factor)	−1,62	0,000256
*MACF1*	Microtubule-actin cross-linking factor 1	−1,56	0,005851
*MID1*	Midline 1	*−2,11*	*0,000067*
*NCK1*	NCK adaptor protein 1	−1,70	0,000862
*MYPT1*	Protein phosphatase 1, regulatory (inhibitor) subunit 12A	−1,77	0,006053
*ROCK1*	Rho-associated, coiled-coil containing protein kinase 1	−1,61	0,000288
*TIAM1*	T-cell lymphoma invasion and metastasis 1	−1,57	0,00189
*WASF1*	WAS protein family, member 1	−1,56	0,000922

*MAP kinase signalling pathway*			

*CDKN2B*	Cyclin-dependent kinase inhibitor 2B (p15, inhibits CDK4)	−2,05	0,000059
*CDKN2D*	Cyclin-dependent kinase inhibitor 2D (p19, inhibits CDK4)	1,68	0,000229
*E2F1*	E2F transcription factor 1	−1,90	0,002717
*FOS*	*c-fos*	*1,99*	*0,002614*
*MAP2K1*	MEK1	1,89	0,000016
*MAP2K6*	*MKK6*	*−2,37*	*0,002281*
*MAPK7*	ERK4	1,70	0,000529
*MAPK13*	p38delta	1,51	0,000418
*SFN*	Stratifin	1,57	0,000872
*TP53*	Tumour protein p53	*−2,03*	0,000005
